# Pelvic inflammatory disease risk following negative results from chlamydia nucleic acid amplification tests (NAATs) versus non-NAATs in Denmark: A retrospective cohort

**DOI:** 10.1371/journal.pmed.1002483

**Published:** 2018-01-02

**Authors:** Bethan Davies, Katy M. E. Turner, Thomas Benfield, Maria Frølund, Berit Andersen, Henrik Westh, on behalf of the Danish Chlamydia Study, Helen Ward

**Affiliations:** 1 Department of Infectious Disease Epidemiology, School of Public Health, Imperial College London, London, United Kingdom; 2 School of Veterinary Sciences, University of Bristol, Bristol, United Kingdom; 3 Department of Infectious Diseases, Hvidovre Hospital, University of Copenhagen, Hvidovre, Denmark; 4 Institute of Clinical Medicine, University of Copenhagen, Copenhagen, Denmark; 5 Research Unit for Reproductive Tract Microbiology, Bacteria, Parasites & Fungi, Infectious Disease Preparedness, Statens Serum Institut, Copenhagen, Denmark; 6 Department of Public Health Programmes, Central Region, Denmark; 7 Department of Clinical Medicine, Aarhus University, Aarhus, Denmark; 8 Department of Clinical Microbiology, Hvidovre Hospital, University of Copenhagen, Hvidovre, Denmark; World Health Organization, SWITZERLAND

## Abstract

**Background:**

Nucleic Acid Amplification Tests (NAATs) are the recommended test type for diagnosing *Chlamydia trachomatis* (chlamydia). However, less sensitive diagnostic methods—including direct immunofluorescence (IF) and enzyme-linked immunoassay (ELISA)—remain in use in lower resourced settings. We estimate the risk of pelvic inflammatory disease (PID) following undiagnosed infection in women tested with non-NAATs and estimate the health gain from using accurate diagnostic tests.

**Methods and findings:**

We used Denmark’s national Chlamydia Study dataset to extract all chlamydia tests performed in women aged 15–34 years (1998–2001). Tests were categorised as non-NAAT (IF/ELISA) or NAAT and limited to each woman’s first test in the study period. We linked test data to hospital presentations for PID within 12 months from the Danish National Patient Register. The study included 272,105 women with a chlamydia test, just under half (44.78%, *n* = 121,857) were tested using NAATs. Overall, 6.38% (*n* = 17,353) tested positive for chlamydia and 0.64% (*n* = 1,732) were diagnosed with PID within 12 months. The risk of PID following a positive chlamydia test did not differ by test type (NAAT 0.81% [95% CI 0.61–1.00], non-NAAT 0.78% [0.59–0.96]). The risk of PID following a negative test was significantly lower in women tested with NAATs compared to non-NAATs (0.55% [0.51–0.59] compared to 0.69% [0.64–0.73]; adjusted odds ratio (AOR) 0.83 [0.75–0.93]). We estimate that 18% of chlamydia infections in women tested with a non-NAAT were undiagnosed and that the risk of progression from undiagnosed chlamydia infection to PID within 12 months was 9.52% (9.30–9.68). Using non-NAATs could lead to an excess 120 cases of PID per 100,000 women tested compared to using NAATs. The key limitations of this study are under ascertainment of PID cases, misclassification bias in chlamydia and PID exposure status, bias to the association between clinical presentation and test type and the presence of unmeasured confounders (including other sexually transmitted infection [STI] diagnoses and clinical indication for chlamydia test).

**Conclusion:**

This retrospective observational study estimates the positive impact on women’s reproductive health from using accurate chlamydia diagnostic tests and provides further evidence for restricting the use of inferior tests. Women with a negative chlamydia test have a 17% higher adjusted risk of PID by 12 months if they are tested using a non-NAAT compared to a NAAT.

## Abstract 2

In a retrospective cohort study, Bethan Davies and colleagues estimate the impact of non-NAAT testing on risk of subsequently developing pelvic inflammatory disease.

## Author summary

**Why was this study done?:**

- There are a range of different tests that can be used to diagnose chlamydia infection. Each of these tests has a different ability to separate the people who are infected with chlamydia from the people who are not infected.
- If a less sensitive test is used, there is a greater chance that an infection in a person with chlamydia won’t be detected by the test. In this situation, the individual won’t be treated and could develop a complication, such as pelvic inflammatory disease, or pass the infection on to someone else.
- We wanted to test our hypothesis that less sensitive (poorer performing) diagnostic tests leave a higher proportion of women who are infected with chlamydia undiagnosed and that this leads to a greater risk of them developing pelvic inflammatory disease within a year.

**What did the researchers do and find?:**

- We used national administrative healthcare data from Denmark to identify women who were tested for chlamydia during the period 1998–2001 when less sensitive chlamydia diagnostic tests (non-NAATs) were gradually replaced by more accurate tests (NAATs). We added information about any hospital presentations (inpatient, outpatient, or Emergency Department) with pelvic inflammatory disease that women had within a year of their chlamydia test.
- We used this dataset to compare the proportion of women who went on to be diagnosed with pelvic inflammatory disease by the type of chlamydia test that they were given (non-NAAT or NAAT).
- There were 272,105 women in this study; 6.4% were diagnosed with chlamydia and 0.6% were diagnosed with pelvic inflammatory disease.
- Women who had a positive test and were diagnosed with chlamydia (and presumably given appropriate treatment for chlamydia) had an equal chance of developing pelvic inflammatory disease regardless of the diagnostic test type used. But for women who had a negative test and were not diagnosed with chlamydia, the chance of developing pelvic inflammatory disease was lower for those who received a more accurate diagnostic test.
- We estimate that almost 1 in 5 women who were infected with chlamydia at the time of their test, but were tested using the less sensitive non-NAATS, went undiagnosed. This could lead to an excess 120 cases of PID per 100,000 women tested with a non-NAAT.

**What do these findings mean?:**

- This study has quantified the positive impact on women’s reproductive health from using accurate chlamydia diagnostic tests and provides further evidence for restricting the use of inferior tests.

## Introduction

Sexually transmitted *C*. *trachomatis* (chlamydia) is the most prevalent sexually transmitted infection (STI) with an estimated 68,455,000 incident cases globally in women in 2012 [1,2]. Chlamydia is the subject of intensive control efforts in many high-income settings [3]. The aim of diagnosing and treating chlamydia is 2-fold: to reduce the risk of progression to complications in the individual (including pelvic inflammatory disease [PID]) and to reduce the risk of transmission to another individual (including neonates).

The method of chlamydia diagnosis has advanced over time. Antigen-based diagnostic tests were introduced in the 1980s to replace culture [4]. Some of these antigen-based tests were relatively labour intensive and nucleic acid amplification tests (NAATs) were developed and increasingly robotized to increase the volume of tests that could be undertaken. The additional advantage of NAATs is that they have a real-world sensitivity of 90%–96%, which leads to a lower proportion of untreated infections (false negative tests) compared to antigen-based methods (direct immunofluorescence (IF) or direct fluorescent antibody (DFA) and enzyme-linked immunosorbent assay (ELISA) or enzyme immunoassay (EIA) sensitivity 65%–75% compared to NAAT) [5,6].

To minimise the risk of undertreatment due to false negative tests, NAATs have been the recommended test type for the diagnosis of chlamydia since the early 2000s [7–9]. However, antigen-based methods remain in use in many settings, often due to resource constraints. They are also widely available for purchase online and sold over the counter in pharmacies, including for home testing [3]. The cost and availability of ASSURED (affordable, sensitive, specific, user-friendly, rapid and robust, equipment-free, and delivered to end-users) diagnostic tests also hampers the implementation of chlamydia case management guidelines in low- and middle-income settings [2]. The WHO recommends syndromic management of vaginal discharge for low-resource settings and this has been widely implemented where expensive diagnostic tests are not available [10–13].

There are important health consequences from less sensitive diagnostic tests and syndromic diagnosis. Infected people with false negative tests are falsely reassured, undiagnosed, untreated, and remain at risk of complications and onward transmission of chlamydia whilst uninfected people with false positive tests are incorrectly diagnosed and given unnecessary treatment.

We hypothesise that the increased risk of undiagnosed chlamydia infection following the use of non-NAATs will lead to an observable higher risk of PID in women who test negative using non-NAATs compared to NAATs. We aim to estimate the risk of PID following an undiagnosed chlamydia infection in women tested with a non-NAAT.

## Methods

### Ethics statement

The Danish Chlamydia Study was approved by the Danish Data Protection Agency (J.nr. 2010-41-4866, J.nr. 2012-331-0228 and J.nr. 2015-41-4344).

### Study design

The Danish Chlamydia study is a purpose-generated dataset of all chlamydia tests performed in public health laboratories in Denmark (including Greenland) between 1st January 1992 and 2nd November 2011. A full description of this dataset has been previously published [14]. For the present study we extracted all chlamydia test records from women aged 15–34 years that were performed between 1st January 1998 and 31st December 2001, the interval when non-NAATs were replaced by NAATs as the most common test type (S1 Fig). In Denmark, the contribution of NAATs to all non-NAAT/NAAT chlamydia tests was 23.5% in 1998 and 66.01% in 2001, and there were 696,987 female residents aged 15–34 years in 2000 [15]. Clinical Microbiology Laboratories (CMLs) switched from non-NAATs to NAATs at individually determined times, with the change-over occurring during the study period for most CMLs. The choice of chlamydia test was made by the CML not by the requesting clinicians and there were no changes to recommendations for chlamydia testing during the study period.

We excluded chlamydia tests that: (a) had an ambiguous result (defined as not positive or negative e.g., “inconclusive” or missing data) or (b) used a test type other than NAAT (defined as PCR, SDA, TMA, LCR, DNA, DNA/RNA) or non-NAAT (defined as ELISA, IF, “antigen”) (examples of excluded test types: unknown, microscopy, culture). We then limited the dataset to the first chlamydia test per woman in this time interval (the index test), preferentially selecting positive tests and NAATs if multiple tests were performed on the same date.

We linked chlamydia test records to hospital healthcare records from the Danish National Patient Register (1993–2012) using the study ID number, which is anonymously linked to the unique Danish patient identification number (key held securely and not accessible to researchers) that is recorded in all administrative healthcare records [16]. Each woman’s first recorded healthcare presentation (outpatient, emergency department, and inpatient) for PID was identified (defined as International Classification of Diseases version 10 (ICD-10) A18·1; A51·4; A52·7; A54·2; A56·1; N70-74·8, ICD-10 coding introduced in Denmark in 1994). Episodes of PID were defined as “previous” if they occurred before the index chlamydia test, “same day” if they occurred on the same date as the index chlamydia test, and “12 months” if they occurred between one and 365 days after the index test. We excluded women who had a history of PID before their index chlamydia test and women who were diagnosed with PID on the same day as their index test.

We defined a priori exposure categories as follows: age at chlamydia test (15–24 years; 25–34 years), year of chlamydia test (1998/1999 and 2000/2001) and chlamydia test positivity (yes/no). Following peer review, we defined two additional explanatory variables: location of the laboratory processing the chlamydia test, defined as (a) STI clinic within their catchment (yes/no), (b) “no” STI clinic as a single group with “yes” STI clinic divided into the five separate laboratories; repeat chlamydia test, defined using the method outlined above and limited to tests performed ≥30 and ≤365 days after the index test and ≤1 day before a PID diagnosis categorised as no/negative/positive. We also categorised age and year into multiple categories and as continuous variables.

### Statistical analysis

For the cohort overall and by chlamydia test type we describe age, year, chlamydia test positivity, laboratory area, repeat chlamydia test, and incidence of PID by 12 months. We used Chi-squared tests to compare the proportion of women in each category by chlamydia test type.

We estimated the risk of PID following an undiagnosed chlamydia infection in women tested using a non-NAAT. To do this, we estimated “i” the proportion of women tested with a non-NAAT who had an undiagnosed infection, under the assumption that NAAT positivity is equal to true population chlamydia positivity (where i = [NAAT positivity] − [non-NAAT positivity]) and “j” the excess proportion of women tested with a non-NAAT who were diagnosed with PID (where j = [risk of PID following a non-NAAT]–[risk of PID following a NAAT]). We then make the assumption that all the observed excess cases of PID in women tested with a non-NAAT (j) occurred in the proportion with an undiagnosed chlamydia infection (i) to estimate “p” the risk of PID following an undiagnosed chlamydia infection in women tested using a non-NAAT (where p = [j]/[i]). Finally we estimate “r”, the number of excess cases of PID that would be observed per 100,000 women tested using a non-NAAT compared to a NAAT (where r = [i]*[p]*100,000).

We used logistic regression to determine the association between chlamydia test type and PID by 12 months adjusted for age, year, and laboratory area. We repeated the analysis stratified by chlamydia test result due to the known difference in the performance of NAAT and non-NAAT diagnostic tests. For each sub-cohort, we compared the risk of PID at 12 months by test type using a difference between two proportions test and adjusted logistic regression analysis. We then repeated this logistic regression analysis including repeat chlamydia test as an explanatory variable. PID is a relatively rare event in this cohort, therefore we refer to the resulting odds of PID as “risks” in the results and discussion because probability is a more natural measure.

## Results

**Fig 1:**
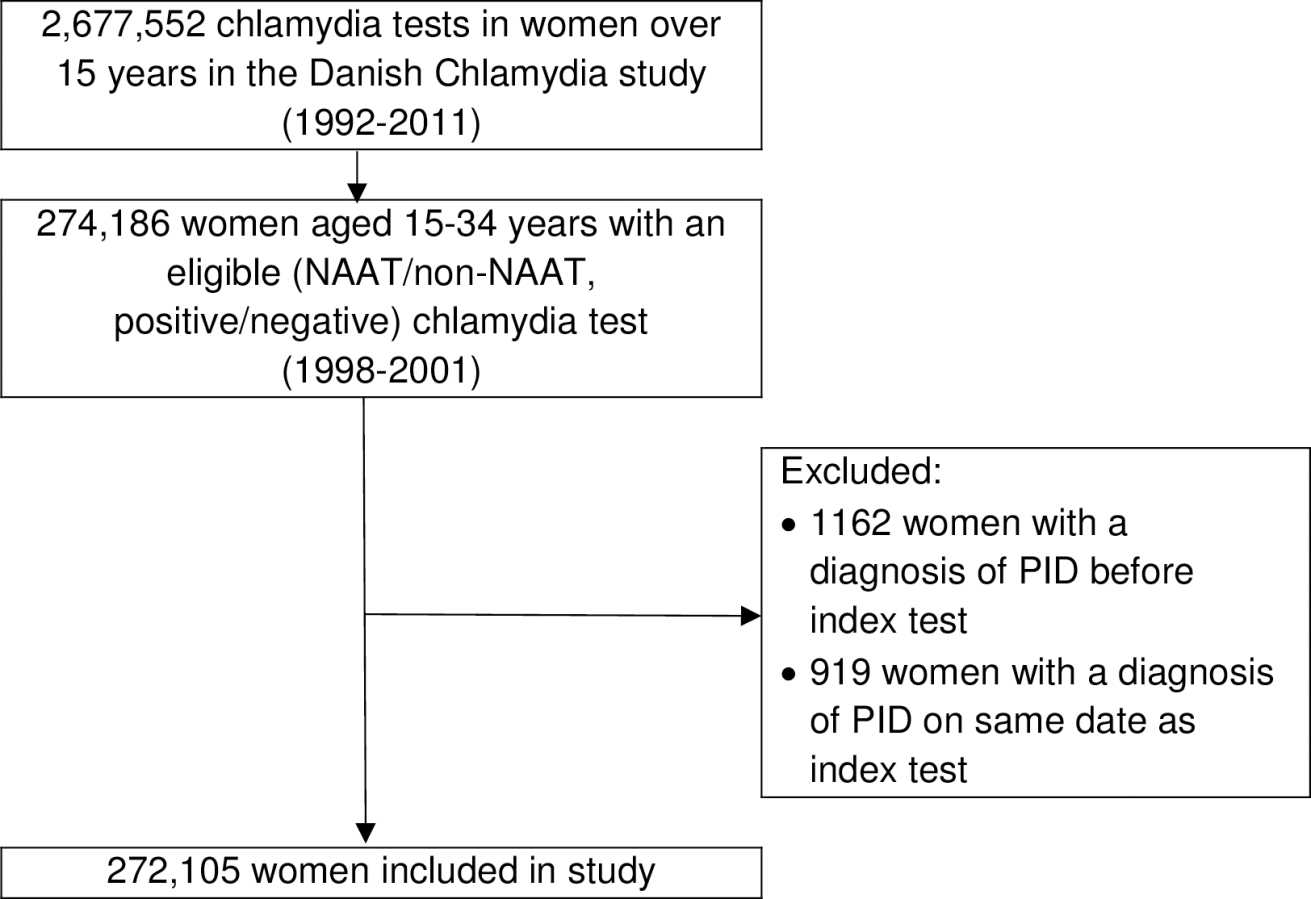
Cohort formation. NAAT, Nucleic Acid Amplification Test; PID, pelvic inflammatory disease.

Generation of the study dataset is illustrated in Fig 1. There were 272,105 women resident in Denmark aged 15–34 years who had an eligible chlamydia test during the study period (1998–2001) and no documented previous history of PID. The mean age of women on the date of their index chlamydia test (i.e., at entry to the study cohort) was 24.80 years (standard deviation 5.06, range 15–34 years). Under half of this cohort were tested using a NAAT (*n* = 121,857, 44.78%), and overall, chlamydia test positivity was 6.38% (*n* = 17,353) (Table 1). A repeat chlamydia test was performed in 61,890 women, of which 5.63% (*n* = 3,484) were positive. The five laboratories with an STI clinic in their catchment area performed 76.20% (*n* = 207,331) of the index chlamydia tests. Two of these five laboratories did not perform non-NAATs during the study period (S1 Table). Compared to women tested using a non-NAAT, women tested using a NAAT were more likely to be younger, tested after 1998, tested by laboratories in areas without an STI clinic, to have a positive index test, and to have a positive repeat test (Table 1).

**Table 1:** Description of the study cohort (1998–2001) by chlamydia test type.

		Overall		Chlamydia test type				Chi-squared*
				Non-NAAT		NAAT		
		*n*	%	*n*	% (95% CI)*	*n*	% (95% CI)~	
**Overall**		272,105		150,248		121,857		
**Age group (years)**	15–24	134,971	49.60	70,786	47.11 (46.86–47.37)	64,185	52.67 (52.39–52.95)	
	25–34	137,134	50.40	79,462	52.89 (52.63–53.14)	57,672	47.33 (47.05–47.61)	<0.001
****Year****	1998	87,365	32.11	66,857	44.50 (44.25–44.75)	20,508	16.83 (16.62–17.04)	
	1999	68,737	25.26	43,600	29.02 (28.79–29.25)	25,137	20.63 (20.40–20.86)	
	2000	62,760	23.06	23,147	15.41 (15.22–15.59)	39,613	32.51 (32.24–32.77)	
	2001	53,243	19.57	16,644	11.08 (10.92–11.24)	36,599	30.03 (29.78–30.29)	<0.001
**STI clinic in laboratory area**	No	64,774	23.80	31,409	20.90 (20.70–21.11)	33,365	27.38 (27.13–27.63)	
	Yes	207,331	76.20	118,839	79.10 (78.89–79.30)	88,492	72.62 (72.37–72.87)	<0.001
**Chlamydia result**	Negative	254,752	93.62	141,516	94.19 (94.07–94.31)	113,236	92.93 (92.78–93.07)	
	Positive	17,353	6.38	8,732	5.81 (5.69–5.93)	8,621	7.07 (6.93–7.22)	<0.001
**Repeat chlamydia test**	No	210,215	77.26	116,306	77.41 (77.20–77.62)	93,909	77.06 (76.83–77.30)	
	Negative	58,406	21.46	32,210	21.44 (21.23–21.65)	26,196	21.50 (21.27–21.73)	
	Positive	3,484	1.28	1,732	1.15 (1.10–1.21)	1,752	1.44 (1.37–1.51)	
**PID by 12 months**	No	270,373	99.36	149,208	99.31 (99.27–99.35)	121,165	99.43 (99.39–99.47)	
	Yes	1,732	0.64	1,040	0.69 (0.65–0.73)	692	0.57 (0.53–0.61)	<0.001

**Abbreviations:** PID, pelvic inflammatory disease; STI, sexually transmitted infection

*% of all non-NAATs; ~ % of all NAATs; ^ comparison by test type of 15-24/over 25; 1998, 1999, 2000, 2001; STI clinic in laboratory area yes/no; chlamydia result positive/negative; repeat chlamydia test no/negative/positive, PID yes/no

**Fig 2:**
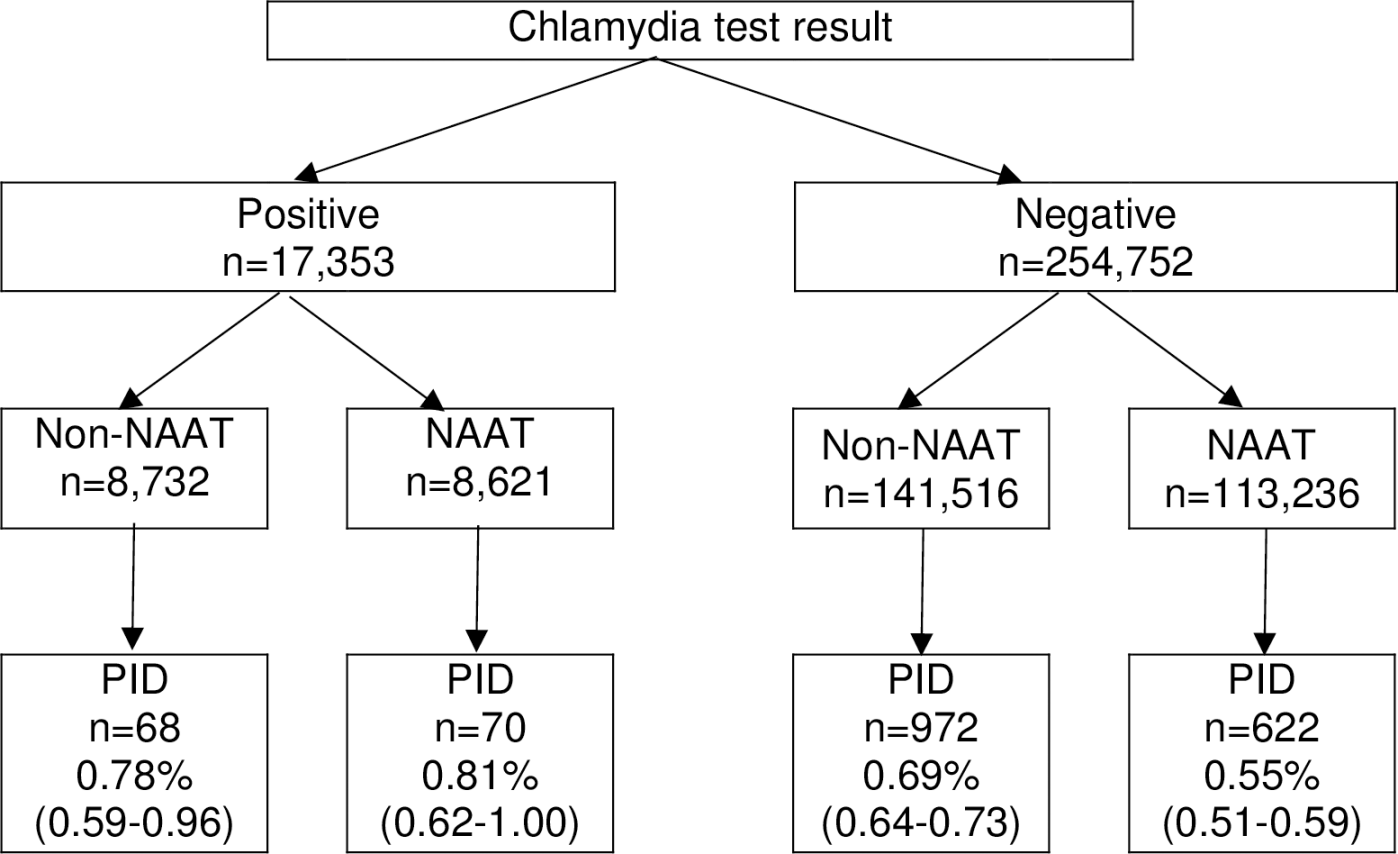
Crude risk of PID by 12 months by test result and test type. NAAT, Nucleic Acid Amplification Test; PID, pelvic inflammatory disease.

Overall, 1,732 (0.64%) women had a hospital healthcare presentation for PID within 12 months of their chlamydia test. Over half (52.19% [*n* = 904]) of women diagnosed with PID were inpatients, 25.40% (*n* = 440) were outpatients and 20.44% (*n* = 354) were treated in emergency departments (the location was unknown for 1.96% [*n* = 34]). There was no difference in the proportion of women with a positive test who progressed to PID within 12 months by test type (0.78% non-NAAT and 0.81% NAAT, *p* = 0.805, Fig 2), but a higher proportion of women progressed to PID following a negative non-NAAT compared to a negative NAAT (0.69% non-NAAT and 0.55% NAAT, *p* < 0.001).

Test positivity was 18% higher following a NAAT compared to a non-NAAT (absolute difference 1.26%, equal to 7.07% minus 5.81%). Therefore, compared to women tested with a NAAT, we estimate that 1.26% (equal to the difference in test positivity) of women tested using a non-NAAT had an undiagnosed chlamydia infection and an additional 0.12% (equal to 0.69% minus 0.57%) were diagnosed with PID. Therefore, the estimated risk of progression from undiagnosed chlamydia infection to PID within 12 months is 9.52% (95% CI 9.30–9.68), and there would be an estimated 120 excess cases of PID per 100,000 women tested with a non-NAAT compared to a NAAT.

The unadjusted risk of PID within 12 months of a chlamydia test was significantly higher in women tested with a non-NAAT, tested in 1998 compared to 2001, aged over 25, and with a positive rather than a negative index test (odds ratio [OR] 1.27 [95% CI 1.07–1.52]) (Table 2). The unadjusted risk of PID in the overall cohort became significantly higher at age 29 (S2 Table). The adjusted risk of PID was 32% higher in women over 25 compared with under 25 years (AOR 1.32 [1.20–1.46]), and 14% lower in women tested in 2001 compared with 1998 (AOR 0.86 [0.74–1.00]) and following a NAAT rather than a non-NAAT (AOR 0.86 [0.78–0.96]).

**Table 2:** Unadjusted and adjusted logistic regression analysis of PID by 12 months by chlamydia test type, age, year of test and laboratory area, overall, and stratified by chlamydia status.

		Number of women	Women with PID at 12 months	Unadjusted logistic regression			Adjusted logistic regression		
				OR	95% CI	*p*-value	AOR	95% CI	*p*-value
**a. Overall**									
**Chlamydia test type**	Non-NAAT	150,248	1,040						
	NAAT	121,857	692	0.82	0.74–0.90	<0.001	0.86	0.78–0.96	0.005
**Age group (years)**	15–24	134,971	735						
	25–34	137,134	997	1.34	1.22–1.47	<0.001	1.32	1.20–1.46	<0.001
**Chlamydia test year**	1998	87,365	579						
	1999	68,737	488	1.07	0.95–1.21	0.261	1.08	0.96–1.23	0.191
	2000	62,760	382	0.92	0.81–1.04	0.195	0.97	0.85–1.11	0.687
	2001	53,243	283	0.80	0.69–0.92	0.002	0.86	0.74–1.00	0.046
**STI clinic in laboratory area**	No	64,774	428						
	Yes	207,331	1,304	0.95	0.85–1.06	0.374	0.91	0.82–1.02	0.114
**Chlamydia test result**	Negative	254,752	1,594						
	Positive	17,353	138	1.27	1.07–1.52	0.007	not included in model		
**b. Chlamydia negative**									
**Chlamydia test type**	Non-NAAT	141,516	972						
	NAAT	113,236	622	0.80	0.72–0.88	<0.001	0.83	0.75–0.93	0.001
**Age group (years)**	15–24	121,996	637						
	25–34	132,756	957	1.38	1.25–1.53	<0.001	1.37	1.24–1.52	<0.001
**Chlamydia test year**	1998	82,458	538						
	1999	64,532	444	1.05	0.93–1.20	0.406	1.07	0.94–1.21	0.299
	2000	58,398	354	0.93	0.81–1.06	0.281	0.99	0.86–1.14	0.934
	2001	49,364	258	0.80	0.69–0.93	0.003	0.87	0.74–1.01	0.075
**STI clinic in laboratory area**	No	60,008	396						
	Yes	194,744	1,198	0.93	0.83–1.04	0.224	0.89	0.80–1.00	0.055
**c. Chlamydia positive**									
**Chlamydia test type**	Non-NAAT	8,732	68						
	NAAT	8,621	70	1.04	0.75–1.46	0.805	1.22	0.85–1.75	0.281
**Age group (years)**	15–24	12,975	98						
	25–34	4,378	40	1.21	0.84–1.75	0.308	1.19	0.82–1.73	0.351
**Chlamydia test year**	1998	4,907	41						
	1999	4,205	44	1.25	0.82–1.92	0.298	1.24	0.80–1.90	0.338
	2000	4,363	28	0.77	0.47–1.24	0.280	0.72	0.43–1.19	0.202
	2001	3,879	25	0.77	0.47–1.27	0.304	0.72	0.43–1.23	0.232
**STI clinic in laboratory area**	No	4,766	32						
	Yes	12,587	106	1.26	0.84–1.87	0.259	1.23	0.83–1.84	0.308

Abbreviations: AOR, adjusted odds ratio; OR, odds ratio; PID, pelvic inflammatory disease; STI, sexually transmitted infection

Stratifying the analysis by test result demonstrated that for women with a positive test (presumably treated) there was no difference in the risk of PID by 12 months by test type (AOR 1.22 [0.85–1.75]). However, women with a negative test (presumably untreated) had a 17% lower risk of PID following a NAAT compared to a non-NAAT (AOR 0.83 [0.75–0.93]).

In a further stratified model including the first repeat chlamydia test within 12 months, women with a positive index test had a 62% lower risk of PID following a negative repeat test compared to women without a repeat test (AOR 0.38 [0.26–0.57]) (S3 Table). For women with a negative index test, having a repeat test was not significantly associated with PID.

## Discussion

Women with a negative chlamydia test had a 17% higher adjusted risk of PID (absolute difference 0.14% [0.69% compared to 0.55%]) by 12 months if they were tested using a non-NAAT compared to a NAAT, but there was no difference in the risk of PID following a positive chlamydia test, supporting our hypothesis. This is presumably due to the higher proportion of false negative tests from less sensitive non-NAATs and we estimate that 18% of chlamydia infections in women tested with a non-NAAT went undiagnosed. This study suggests that the risk of progression to PID following an untreated infection was 9.52%. We quantify the health impact of using less sensitive chlamydia diagnostic tests as an excess 120 cases of PID per 100,000 women tested, which provides further evidence for restricting the use of non-NAATs and other less sensitive diagnostic tests.

This is the first published comparison of the risk of PID following chlamydia testing that takes into account diagnostic test type. There are several factors that have the potential to contribute to the observed excess risk of PID following a negative non-NAAT compared to a NAAT. Firstly, a higher proportion of women tested with non-NAATs were from the older age group compared to the younger age group. PID risk is known to increase with age but the increased risk of PID remained after adjustment for age [17]. Secondly, non-NAATs were more common in the earlier time interval but it is unlikely that there were PID risk factors (e.g., prevalence of non-chlamydia causes of PID) specific to this period that resolved by 2001 and the association remained in the adjusted analysis. Thirdly, there is the potential for bias in the application of chlamydia diagnostic test type by PID risk or other unmeasured confounders. However, the choice of diagnostic test was undertaken in the laboratory (not by the clinician), and each laboratory usually changed from non-NAATs to NAATs at a discrete time as machinery was replaced, therefore with only a short test overlap until the old test was closed down. The association remained after adjustment for the presence of an STI clinic in the catchment of laboratories.

Therefore, the most likely explanation for a difference in the risk of PID by test type following a negative chlamydia test is the difference in test performance and the proportion of undiagnosed infections. In this cohort, chlamydia test positivity was 18% higher in women tested with a NAAT compared to those tested with a non-NAAT (7.07% compared to 5.81%), which is broadly in keeping with the reported 65%–75% sensitivity of non-NAATs compared to NAATs [5,6].

NAATs are universally recommended for the diagnosis of chlamydia in sexually active adults but their use is infrequent in a significant minority of European countries (*n* = 9/28) [3]. Data collected from 28 European countries in 2012 by the European Centre for Disease Prevention and Control found that NAATs were unavailable in the public sector in five countries and at least four countries used non-NAATs for the majority of chlamydia diagnostic tests. It is likely that the higher price of NAATs compared to non-NAATs is at least partly contributing to the reported deviation from international guidance. In addition to use by public-sector authorities, EIA chlamydia tests are easily available for purchase online and in-person in many countries. It can be difficult to access information about the test, including its performance, which raises challenging questions about the regulation of online diagnostic tests.

Syndromic management of vaginal discharge is widely applied in low-resource settings where diagnostic tests are unavailable [13]. This approach generally leads to an overdiagnosis of chlamydia in uninfected women and undertreatment of those who are asymptomatic (estimated to be 80% of all infections) [2]. A recent expert commission supports the development of point-of-care tests (POCTs) for use in low-resource settings as a major player in the pathway to improved chlamydia control [2]. But current efforts are hampered by the poor performance of POCTs (sensitivity in women 22.7%–93.8%, specificity 89.0%–100%) [2]. The WHO is planning to develop guidance on STI laboratory diagnosis and screening in 2017/2018. We hope that this will address the challenge of balancing the risks of using less sensitive diagnostic tests against syndromic management approaches in the context of resource availability and chlamydia control impact [9].

Of further relevance to chlamydia control policy, our supplementary analysis suggests that the age at which the risk of PID increased was 29 years, which is older than the cutoff (25 years) commonly used in chlamydia testing guidelines. This interesting finding warrants further exploration.

In this analysis we made crude assumptions about the underlying prevalence of chlamydia infection and the number of women at risk of PID in the non-NAAT group to estimate that the risk of progression to PID following an undiagnosed infection was 9.52% (9.30–9.68). This estimate is remarkably consistent with the most robust observed risk of 9.46% (2.79–16.13) from the Prevention Of Pelvic Infection (POPI) Randomised Controlled Trial (RCT) [18]. This similarity in result would suggest that the estimates are approximating a true underlying biological risk. However, cohort studies of the risk of complications following chlamydia infection are hampered by the unknown impact of unmeasured confounders including undiagnosed repeat chlamydia infections, other incident STIs, and diagnostic biases. A further methodological challenge is that all subsequent diagnoses of PID within the timeframe are attributed to the incident chlamydia infection, which is likely to overestimate the true biological association. It is important to acknowledge that some of these limitations can be considered to apply equally within the POPI RCT as women were not repeatedly tested for STIs during follow-up.

We agree that parameter estimates obtained from cohort studies should be interpreted cautiously. However, this is the second example of the Danish Chlamydia Study producing estimates of the risk of complications following chlamydia that are consistent with other study designs. In a recent analysis, our estimate of the population excess fraction of chlamydia on PID was consistent with estimates from a multi-parameter evidence synthesis [19]. We suggest that the consistency between our estimates and those of others support the view that cohort studies are a valid and informative study design in this context.

For completeness, we should consider that NAATs are not without their critics. Hadgu and Sternberg argue that the true specificity of NAATs is lower than that commonly reported and that the risk of progression to PID is lower following NAATs compared to other diagnostic test types because NAATs can detect small quantities of genetic material that may be associated with infection of lower pathogenic potential [20]. Our study does not support this hypothesis as we found no difference in the risk of progression to PID following a positive diagnostic test (NAAT 0.81% [0.62–1.00] versus non-NAAT 0.78% [0.59–0.96]).

The data used in this study was drawn from an unselected dataset that contains complete ascertainment of chlamydia tests performed in public laboratories in Denmark (1992–2011). There are no private microbiology laboratories in Denmark, therefore the study cohort is representative of the national population of women aged 15–34 years who were tested for chlamydia. However, within this representative cohort, there is the potential for a systematic difference in the clinical risk profile of women tested by CML because only five CMLs had an STI clinic in their catchment at the time of the study. These CMLs are situated in large metropolitan areas. In the study dataset the proportion of tests performed using a NAAT varied from 5.5% to 100% for the CMLs with STI clinics in their catchment, but as a group this proportion was more similar to those observed in CMLs without STI clinics and the overall cohort (42.7%; 51.5%, and 44.8% respectively). We are not able to explore this potential bias further because data on the source of the chlamydia test (e.g., STI clinic or primary care) or the clinical indication for the test (e.g., symptomatic or asymptomatic) is not available. To control for potential confounding between test type and PID, CML was included in the adjusted analysis.

We applied exclusion criteria to improve the accuracy of chlamydia exposure categorisation. The coding in the original laboratory data did not allow identification of specific manufacturers’ tests, therefore we categorised chlamydia test type broadly into NAAT and non-NAAT (excluding culture and microscopy). Our analysis assumes a homogenous performance of diagnostic tests within a category.

A four-year time interval that spanned the introduction of NAATs in Denmark was chosen to maximise the study population size whilst limiting the potential for other relevant secular changes. The study cohort represents approximately 39.0% of Denmark’s female population, 15–34 years (*n* = 696,987 on 1st January 2000) [15,21]. Generalisation of the findings from this analysis to the contemporary setting may be compromised if intervening updates to chlamydia testing policies have led to a change in the composition (demographic or risk) of the population being tested for chlamydia.

We used a broad ICD-10 definition of PID that is consistent with comparable research. The ICD-10 codes included in the final dataset were all within the range N70–74.4. Healthcare presentations with a diagnostic code for PID were obtained from the hospital setting (emergency department, outpatient and inpatient). Data on cases of PID that were only seen in primary/community care were not available, therefore our estimates of the absolute risk of PID following chlamydia are likely to underestimate the true risk. We consider that the PID events included in this study will represent the more severe disease in the population and therefore potentially the most significant events in terms of future reproductive health. The incomplete ascertainment is unlikely to be biased by chlamydia test type. We excluded women who were diagnosed with PID on the same date as their index chlamydia test as this is independent of chlamydia test type. We also excluded women with a past history of PID to reduce any potential biases in future risk.

Chlamydia exposure status in this cross-sectional study is based on a single diagnostic test result and we assume that all diagnosed infections were treated. We did not apply exclusion criteria based on women’s previous history of chlamydia testing or infection. We assume that the distribution of both diagnosed and undiagnosed previous chlamydia infection (before 1998) would be independent of chlamydia test type.

This analysis also assumes that there is a causal relationship between the index chlamydia infection (diagnosed or undiagnosed) and subsequent PID. To improve this assumption, we limited episodes of PID to those within 12 months of the chlamydia test as the estimated duration of an untreated chlamydia infection is in the order of 15 months and RCTs report the risk of PID at 12 months after the test [22,23]. This assumption is likely to overestimate the measured strength of association. We were able to adjust the analysis for test year, age, laboratory area, and repeat chlamydia test but data on other confounders were not available (e.g., indication for the chlamydia test (symptomatic or asymptomatic); antibiotic use; individual level incidence of other causes of PID (e.g., other STIs); healthcare seeking behaviour). It is unlikely that these confounders are associated with diagnostic test type.

## Conclusion

NAATs have been the recommended test type for the diagnosis of chlamydia in sexually active adults for over a decade due to their superior performance over non-NAATs. We found that women with a negative chlamydia test had a 17% higher risk of PID by 12 months if they were tested using a non-NAAT compared to a NAAT and estimate that using non-NAATs will result in an additional 120 cases of PID per 100,000 tested women. This is presumably due to the higher proportion of false negative tests. This study has quantified the positive impact on women’s reproductive health from using accurate chlamydia diagnostic tests and provides further evidence for restricting the use of inferior non-amplified assays including antigen-based diagnostic tests.

## Supporting information

S1 Fig Chlamydia tests in Denmark by test type (NAAT and non-NAAT), 1991–2009. NAAT, Nucleic Acid Amplification Test.
(TIF)

S1 Table Description of chlamydia test type in the overall cohort by age at test (single year categories) and laboratory area (six categories). (PDF)

S2 Table Unadjusted and adjusted logistic regression analysis of PID by 12 months by chlamydia test type, age (single year categories), year of test, and laboratory area, overall. PID, pelvic inflammatory disease.
(PDF)

S3 Table Unadjusted and adjusted logistic regression analysis of PID by 12 months by chlamydia test type, age, year of test, and laboratory area and repeat chlamydia test, overall and stratified by chlamydia status. PID, pelvic inflammatory disease.
(PDF)

S4 Table STROBE checklist. (PDF)

## Abbreviations

AOR: adjusted odds ratio
ASSURED: affordable, sensitive, specific, user-friendly, rapid and robust, equipment-free, and delivered to end-users
CML: Clinical Microbiology Laboratory
DFA: direct fluorescent antibody
EIA: enzyme immunoassay
ELISA: enzyme-linked immunoassay
ICD-10: International Classification of Diseases version 10
IF: immunofluorescence
NAAT: Nucleic Acid Amplification Test
PID: pelvic inflammatory disease
POCT: point-of-care test
POPI: Prevention Of Pelvic Infection
RCT: randomised controlled trial
STI: sexually transmitted infection
