# Supplementary figures and images for: Pelvic inflammatory disease risk following negative results from chlamydia nucleic acid amplification tests (NAATs) versus non-NAATs in Denmark: A retrospective cohort
Source: PLoS Med. 2018 Jan 2;15(1):e1002483. doi: 10.1371/journal.pmed.1002483 (PMC5749678; doi:10.1371/journal.pmed.1002483)

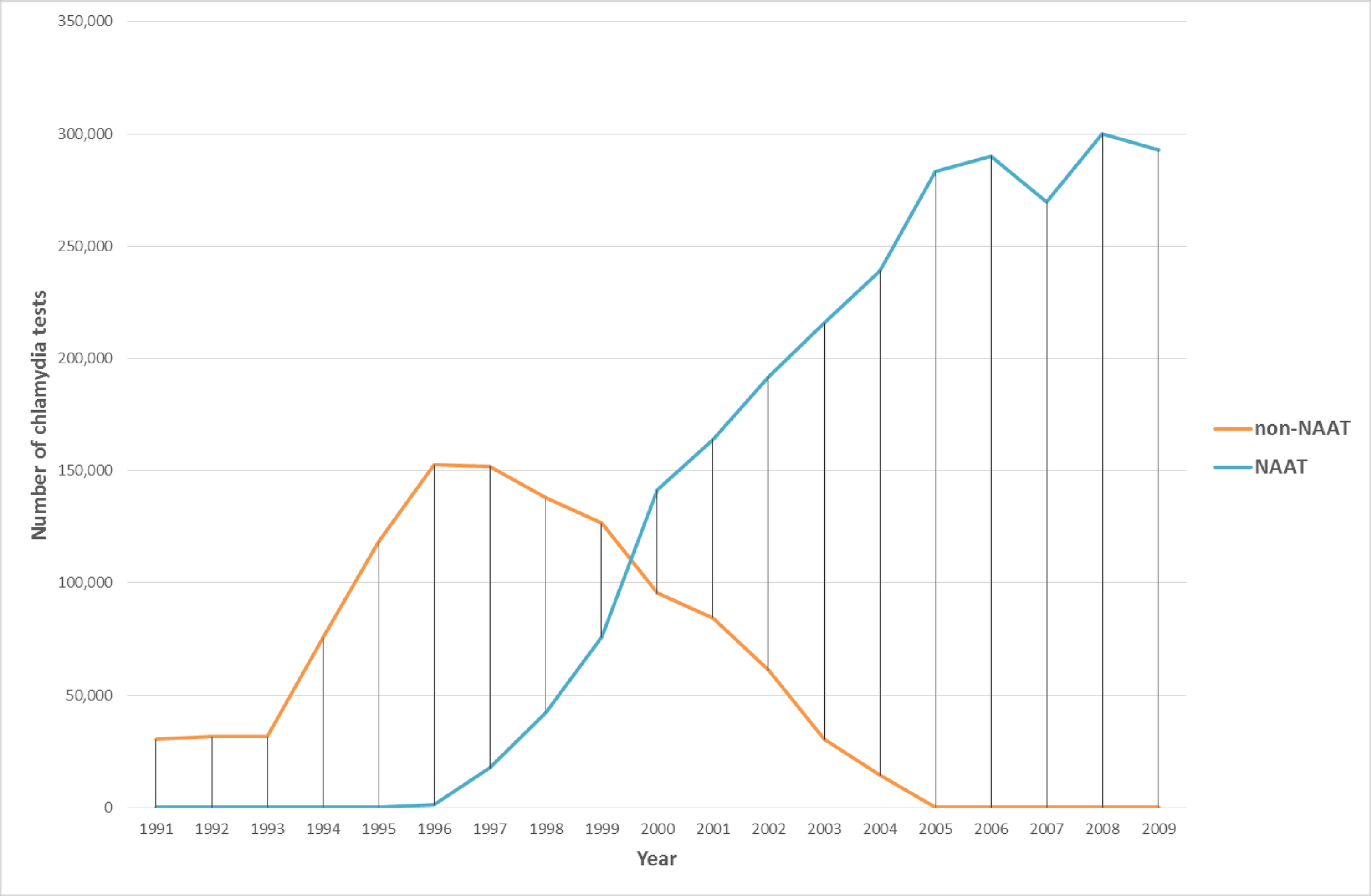

Supplement: S1 Fig — NAAT, Nucleic Acid Amplification Test. (TIF) [file pmed.1002483.s001.tif]
