# Supplementary material for: Pelvic inflammatory disease risk following negative results from chlamydia nucleic acid amplification tests (NAATs) versus non-NAATs in Denmark: A retrospective cohort
Source: PLoS Med. 2018 Jan 2;15(1):e1002483. doi: 10.1371/journal.pmed.1002483 (PMC5749678; doi:10.1371/journal.pmed.1002483)
Supplement: S1 Table — (PDF) [file pmed.1002483.s002.pdf]

S1 Table: Description of chlamydia test type in the overall cohort by age at test (single year categories) and laboratory area (six categories).

			Overall		Chlamydia test type				Chi-squared^
					Non-NAAT		NAAT		
			n	%	n	% (95% CI)*	n	% (95% CI)~	
**Age (years)**	15		4,198	1.54	1,731	1.15 (1.10-1.21)	2,467	2.02 (1.95-2.11)	
	16		7,276	2.67	3,156	2.10 (2.03-2.17)	4,120	3.38 (3.28-3.48)	
	17		9,706	3.57	4,337	2.89 (2.80-2.97)	5,369	4.41 (4.29-4.52)	
	18		11,812	4.34	5,581	3.71 (3.62-3.81)	6,231	5.11 (4.99-5.24)	
	19		13,419	4.93	6,646	4.42 (4.32-4.53)	6,773	5.56 (5.43-5.69)	
	20		15,576	5.72	8,088	5.38 (5.27-5.50)	7,488	6.14 (6.01-6.28)	
	21		16,975	6.24	9,219	6.14 6.02-6.26)	7,756	6.36 (6.23-6.50)	
	22		17,887	6.57	9,969	6.64 (6.51-6.67)	7,918	6.50 (6.36-6.64)	
	23		20,914	7.69	11,967	7.96 (7.83-8.10)	8,947	7.34 (7.20-7.49)	
	24		17,208	6.32	10,092	6.72 (6.59-6.84)	7,116	5.84 (5.71-5.97)	
	25		17,421	6.40	10,222	6.80 (6.68-6.93)	7,199	5.91 (5.78-6.04)	
	26		16,967	6.24	9,895	6.59 (6.46-6.71)	7,072	5.80 (5.67-5.93)	
	27		15,421	5.67	9,097	6.05 (5.93-6.18)	6,324	5.19 (5.06-5.32)	
	28		14,683	5.40	8,490	5.65 (5.53-5.77)	6,193	5.08 (4.96-5.21)	
	29		13,617	5.00	7,800	5.19 (5.08-5.30)	5,817	4.77 (4.65-4.89)	
	30		12,790	4.70	7,377	4.91 (4.80-5.02)	5,413	4.44 (4.33-4.56)	
	31		12,479	4.59	7,348	4.89 (4.78-5.00)	5,131	4.21 (4.10-4.32)	
	32		11,990	4.41	6,928	4.61 (4.51-4.71)	5,062	4.15 (4.04-4.27)	
	33		11,292	4.15	6,492	4.32 (4.22-4.42)	4,800	3.94 (3.83-4.05)	
	34		10,474	3.85	5,813	3.87(3.77-3.97)	4,661	3.82 (3.72-3.93)	<0.001
**STI clinic in laboratory area**	No		64,774	23.80	31,409	20.90 (20.70-21.11)	33,365	27.38 (27.13-27.63)	
	Yes	1	34,693	12.75	32,787	21.82 (21.61-22.03)	1,906	1.56 (1.50-1.64)	
		2	73,941	27.17	68,098	45.32 (45.07-45.58)	5,843	4.79 (4.68-4.91)	
		3	32,111	11.80	17,954	11.95 (11.79-12.11)	14,157	11.62 (11.44-11.80)	
		4	15,404	5.66	0	n/a	15,404	12.64 (12.45-12.83)	
		5	51,182	18.81	0	n/a	51,182	42.00 (41.72-42.28)	<0.001

Abbreviations: *NAAT, Nucleic Acid Amplification Test. STI, Sexually Transmitted Infection. *% of all non-NAATs; ~ % of all NAATs; ^comparison by age (15 to 34) and STI clinic in laboratory area (no, 1 to 5)*
