# Supplementary material for: Pelvic inflammatory disease risk following negative results from chlamydia nucleic acid amplification tests (NAATs) versus non-NAATs in Denmark: A retrospective cohort
Source: PLoS Med. 2018 Jan 2;15(1):e1002483. doi: 10.1371/journal.pmed.1002483 (PMC5749678; doi:10.1371/journal.pmed.1002483)
Supplement: S2 Table — PID, pelvic inflammatory disease. (PDF) [file pmed.1002483.s003.pdf]

S2 Table: Unadjusted and adjusted logistic regression analysis of PID by 12 months by chlamydia test type, age (single year), year of test and laboratory area, overall

		Number of women	Women with PID at 12 months	Unadjusted logistic regression			Adjusted logistic regression		
				OR	95% CI	p value	AOR	95% CI	p value
Chlamydia test type	Non-NAAT	150,248	1,040						
	NAAT	121,165	692	0.82	0.74-0.90	<0.001	0.86	0.77-0.95	0.004
Age (year)	15	4,198	17						
	16	7,276	32	1.09	0.60-1.96	0.783	1.07	0.60-1.94	0.814
	17	9,706	53	1.35	0.78-2.33	0.282	1.32	0.77-2.29	0.314
	18	11,812	82	1.72	1.02-2.90	0.042	1.67	0.99-2.83	0.054
	19	13,419	91	1.68	1.00-2.82	0.050	1.63	0.97-2.73	0.066
	20	15,576	83	1.32	0.78-2.22	0.301	1.27	0.75-2.15	0.368
	21	16,975	86	1.25	0.74-2.11	0.398	1.21	0.72-2.04	0.478
	22	17,887	87	1.20	0.71-2.02	0.489	1.16	0.69-1.95	0.586
	23	20,914	113	1.34	0.80-2.23	0.266	1.28	0.77-2.14	0.340
	24	17,208	91	1.31	0.78-2.20	0.311	1.25	0.74-2.10	0.406
	25	17,421	94	1.33	0.80-2.24	0.275	1.27	0.76-2.14	0.362
	26	16,967	103	1.50	0.90-2.51	0.121	1.44	0.86-2.41	0.167
	27	15,421	93	1.49	0.89-2.51	0.130	1.43	0.84-2.39	0.181
	28	14,683	94	1.58	0.94-2.66	0.081	1.52	0.90-2.55	0.114
	29	13,617	100	1.82	1.09-3.05	0.023	1.75	1.04-2.93	0.034
	30	12,790	106	2.06	1.23-3.43	0.006	1.97	1.18-3.29	0.010
	31	12,479	106	2.11	1.26-3.52	0.004	2.01	1.20-3.36	0.008
	32	11,990	106	2.19	1.31-3.67	0.003	2.09	1.25-3.50	0.005
	33	11,292	102	2.24	1.34-3.75	0.002	2.14	1.28-3.59	0.004
	34	10,474	93	2.20	1.31-3.70	0.003	2.12	1.26-3.56	0.004
Chlamydia test year	1998	87,365	579						
	1999	68737	488	1.07	0.95-1.21	0.261	1.08	0.96-1.22	0.202
	2000	62760	382	0.92	0.81-1.04	0.195	0.97	0.85-1.11	0.658
	2001	53243	283	0.80	0.69-0.92	0.002	0.86	0.74-0.99	0.042
STI clinic in laboratory area	No	64,774	428						
	Yes	207,331	1304	0.95	0.85-1.06	0.374	0.93	0.83-1.04	0.188

Abbreviations: *PID, Pelvic Inflammatory Disease. OR, Odds Ratio. CI, Confidence Interval. AOR, Adjusted Odds Ratio. NAAT, Nucleic Acid Amplification Test. STI, Sexually Transmitted Infection.*
