# Supplementary material for: Pelvic inflammatory disease risk following negative results from chlamydia nucleic acid amplification tests (NAATs) versus non-NAATs in Denmark: A retrospective cohort
Source: PLoS Med. 2018 Jan 2;15(1):e1002483. doi: 10.1371/journal.pmed.1002483 (PMC5749678; doi:10.1371/journal.pmed.1002483)
Supplement: S3 Table — PID, pelvic inflammatory disease. (PDF) [file pmed.1002483.s004.pdf]

S3 Table: Unadjusted and adjusted logistic regression analysis of PID by 12 months by chlamydia test type, age, year of test, laboratory area and repeat chlamydia test, overall and stratified by index chlamydia status.

		Number of women	Women with PID at 12 months	Unadjusted logistic regression			Adjusted logistic regression		
				OR	95% CI	p value	AOR	95% CI	p value
**a. Overall**									
Chlamydia test type	Non-NAAT	150,248	1,040						
	NAAT	121,165	692	0.82	0.74-0.90	<0.001	0.86	0.78-0.96	0.005
Age group (years)	15-24	134,971	735						
	25-34	137,134	997	1.34	1.22-1.47	<0.001	1.31	1.19-1.44	<0.001
Chlamydia test year	1998	87,365	579						
	1999	68,737	488	1.07	0.95-1.21	0.261	1.08	0.96-1.22	0.221
	2000	62,760	382	0.92	0.81-1.04	0.195	0.97	0.84-1.11	0.619
	2001	53,243	283	0.80	0.69-0.92	0.002	0.85	0.73-0.99	0.035
STI clinic in laboratory area	No	64,774	428						
	Yes	207,331	1,304	0.95	0.85-1.06	0.374	0.92	0.82-1.03	0.129
Chlamydia test result	Negative	254,752	1,594						
	Positive	17,353	138	1.27	1.07-1.52	0.007	not included in model		
Repeat chlamydia test	No	210,215	1,396						
	Negative	58,406	319	0.82	0.73-0.93	0.002	0.84	0.74-0.95	0.004
	Positive	3,484	17	0.73	0.45-1.18	0.205	0.81	0.50-1.31	0.399

**b. Chlamydia negative**									
Chlamydia test type	Non-NAAT	141,516	972						
	NAAT	113,236	622	0.80	0.72-0.88	<0.001	0.83	0.75-0.93	0.001
Age group (years)	15-24	121,996	637						
	25-34	132,756	957	1.38	1.25-1.53	<0.001	1.36	1.23-1.50	<0.001
Chlamydia test year	1998	82,458	538						
	1999	64,532	444	1.05	0.93-1.20	0.406	1.07	0.94-1.21	0.331
	2000	58,398	354	0.93	0.81-1.06	0.281	0.99	0.86-1.14	0.879
	2001	49,364	258	0.80	0.69-0.93	0.003	0.86	0.74-1.01	0.064
STI clinic in laboratory area	No	60,008	396						
	Yes	194,744	1,198	0.93	0.83-1.04	0.224	0.90	0.80-1.01	0.061
Repeat chlamydia test	No	201,550	1,301						
	Negative	50,693	286	0.87	0.77-0.99	0.039	0.89	0.78-1.01	0.069
	Positive	2,509	7	0.43	0.20-0.91	0.026	0.48	0.23-1.01	0.054

**c. Chlamydia positive**										
Chlamydia test type	Non-NAAT	8,732	68							
	NAAT	8,621	70	1.04	0.75-1.46	0.805	1.23	0.85-1.76	0.272
Age group (years)	15-24	12,975	98						
	25-34	4,378	40	1.21	0.84-1.75	0.308	1.18	0.82-1.72	0.377
Chlamydia test year	1998	4,907	41						
	1999	4,205	44	1.25	0.82-1.92	0.298	1.22	0.79-1.89	0.360
	2000	4,363	28	0.77	0.47-1.24	0.280	0.70	0.42-1.16	0.167
	2001	3,879	25	0.78	0.47-1.27	0.304	0.70	0.41-1.18	0.183
STI clinic in laboratory area	No	4,766	32						
	Yes	12,587	106	1.26	0.84-1.87	0.259	1.27	0.85-1.89	0.249
Repeat chlamydia test	No	8,665	95						
	Negative	7,713	33	0.39	0.27-0.58	<0.001	0.38	0.26-0.57	<0.001
	Positive	975	10	0.93	0.49-1.80	0.840	0.96	0.50-1.86	0.911

Abbreviations: *PID, Pelvic Inflammatory Disease. OR, Odds Ratio. CI, Confidence Interval. AOR, Adjusted Odds Ratio. STI, Sexually Transmitted Infection*
