# Supplementary material for: Pelvic inflammatory disease risk following negative results from chlamydia nucleic acid amplification tests (NAATs) versus non-NAATs in Denmark: A retrospective cohort
Source: PLoS Med. 2018 Jan 2;15(1):e1002483. doi: 10.1371/journal.pmed.1002483 (PMC5749678; doi:10.1371/journal.pmed.1002483)
Supplement: S4 Table — (PDF) [file pmed.1002483.s005.pdf]

STROBE Statement—Checklist of items that should be included in reports of ***cohort studies***

Article: Pelvic inflammatory disease risk following negative results from chlamydia nucleic acid amplification tests (NAATs) versus non-NAATs in Denmark: A retrospective cohort study

	Item No	Recommendation	Notes
**Title and abstract**	1	(*a*) Indicate the study's design with a commonly used term in the title or the abstract	Title: Pelvic inflammatory disease risk following negative results from nucleic acid amplification chlamydia tests (NAAT) versus non-NAATs in Denmark: A retrospective cohort study
		(*b*) Provide in the abstract an informative and balanced summary of what was done and what was found	Abstract
**Introduction**			
Background/rationale	2	Explain the scientific background and rationale for the investigation being reported	Introduction, paragraph 3: "NAATs have been the recommended test type for the diagnosis of chlamydia for over a decade.[10-12] However, antigen-based methods remain in use in many settings".
Objectives	3	State specific objectives, including any prespecified hypotheses	Introduction, paragraph 5: "We hypothesise that the increased risk of undiagnosed chlamydia infection following the use of non-NAATS will lead to an observable higher risk of PID in women who test negative using non-NAATs compared to NAATs. We aim to estimate the risk of PID following an undiagnosed chlamydia infection in women tested with a non-NAAT."
**Methods**			
Study design	4	Present key elements of study design early in the paper	Methods, paragraph 1-3: "The Danish Chlamydia study is a purpose generated dataset of all chlamydia tests performed in public health laboratories in Denmark"; "we extracted all chlamydia test records from women aged 15-34 years that were performed between 1st January 1998 and 31st December 2001"; also detail inclusion/exclusion criteria; "We linked chlamydia test records to hospital healthcare records from the Danish National Patient Register" and detail outcome events and ICD codes.
Setting	5	Describe the setting, locations, and relevant dates, including periods of recruitment, exposure, follow-up, and data collection	All provided in methods section

Participants	6	(*a*) Give the eligibility criteria, and the sources and methods of selection of participants. Describe methods of follow-up	Methods, paragraph 2: "we extracted all chlamydia test records from women aged 15-34 years that were performed between 1st January 1998 and 31st December 2001, the interval when non-NAATs were replaced by NAATs as the most common test type (S1 Fig)."<br><br>Methods paragraph 3-4: "We excluded chlamydia tests that: (a) had an ambiguous result (defined as not positive or negative e.g. "inconclusive" or missing data) or (b) used a test type other than NAAT (defined as PCR, SDA, TMA, LCR, DNA, DNA/RNA) or non-NAAT (defined as ELISA, IF, "antigen") (examples of excluded test types: unknown, microscopy, culture). We then limited the dataset to the first chlamydia test per women in this time interval (the index test), preferentially selecting positive tests and NAATs if multiple tests were performed on the same date. We linked chlamydia test records to hospital healthcare records from the Danish National Patient Register (1993-2012) using the study ID number which is anonymously linked to the unique Danish patient identification number (key held securely and not accessible to researchers) that is recorded in all administrative healthcare records.[16] Each woman's first recorded healthcare presentation (out-patient, emergency department and in-patient) for PID was identified (defined as ICD-10 A18·1; A51·4; A52·7; A54·2; A56·1; N70-74·8, ICD-10 coding introduced in Denmark in 1994). Episodes of PID were defined as "previous" if they occurred before the index chlamydia test, "same day" if they occurred on the same date as the index chlamydia test and "12 months" if they occurred between one and 365 days after the index test. We excluded women who had a history of PID before their index chlamydia test and women who were diagnosed with PID on the same day as their index test."
		(*b*) For matched studies, give matching criteria and number of exposed and unexposed	N/A
Variables	7	Clearly define all outcomes, exposures, predictors, potential confounders, and effect modifiers. Give diagnostic criteria, if applicable	Methods, paragraph 4: "PID (defined as ICD-10 A18·1; A51·4; A52·7; A54·2; A56·1; N70-74·8)"
Data sources/ measurement	8*	For each variable of interest, give sources of data and details of methods of assessment (measurement). Describe comparability of assessment methods if there is more than one group	Details of two administrative datasets provided in methods.
Bias	9	Describe any efforts to address potential sources of bias	N/A

Study size	10	Explain how the study size was arrived at	N/A
Quantitative variables	11	Explain how quantitative variables were handled in the analyses. If applicable, describe which groupings were chosen and why	Methods, paragraph 5: "age (15-24 years and 25-34 years), year of chlamydia test (1998/9 and 2000/1),"…"We also categorised age and year into multiple categories and as continuous variables".
Statistical methods	12	(*a*) Describe all statistical methods, including those used to control for confounding	Methods, paragraph 6/8: chi-squared tests and logistic regression analysis<br>Methods, paragraph 7: bespoke calculations described
		(*b*) Describe any methods used to examine subgroups and interactions	Methods, paragraph 8: cohort divided into chlamydia positive and negative
		(*c*) Explain how missing data were addressed	N/A
		(*d*) If applicable, explain how loss to follow-up was addressed	N/A
		(*e*) Describe any sensitivity analyses	N/A
**Results**			
Participants	13*	(a) Report numbers of individuals at each stage of study—eg numbers potentially eligible, examined for eligibility, confirmed eligible, included in the study, completing follow-up, and analysed	Detailed in Figure 1
		(b) Give reasons for non-participation at each stage	Figure 1
		(c) Consider use of a flow diagram	Figure 1
Descriptive data	14*	(a) Give characteristics of study participants (eg demographic, clinical, social) and information on exposures and potential confounders	Table 1
		(b) Indicate number of participants with missing data for each variable of interest	N/A
		(c) Summarise follow-up time (eg, average and total amount)	12 months for each participant
Outcome data	15*	Report numbers of outcome events or summary measures over time	Table 1
Main results	16	(*a*) Give unadjusted estimates and, if applicable, confounder-adjusted estimates and their precision (eg, 95% confidence interval). Make clear which confounders were adjusted for and why they were included	Table 2
		(*b*) Report category boundaries when continuous variables	Age group; year

		were categorized	
		(*c*) If relevant, consider translating estimates of relative risk into absolute risk for a meaningful time period	Results, paragraph 3: “Test positivity was 18% higher following a NAAT compared to a non-NAAT (absolute difference 1.26%, equal to 7.07% minus 5.81%). Therefore compared to women tested with a NAAT, we estimate that 1.26% (equal to the difference in test positivity) of women tested using a non-NAAT had an undiagnosed chlamydia infection and an additional 0.12% (equal to 0.69% minus 0.57%) were diagnosed with PID. Therefore, the estimated risk of progression from undiagnosed chlamydia infection to PID within 12 months is 9.52% (95% CI 9.30-9.68) and there would be an estimated 120 excess cases of PID per 100,000 women tested with a non-NAAT compared to a NAAT.”
Other analyses	17	Report other analyses done—eg analyses of subgroups and interactions, and sensitivity analyses	N/A
**Discussion**			
Key results	18	Summarise key results with reference to study objectives	Discussion, paragraph 1
Limitations	19	Discuss limitations of the study, taking into account sources of potential bias or imprecision. Discuss both direction and magnitude of any potential bias	Discussion, paragraph 10-15<br>Discussion, paragraph 7: “cohort studies of the risk of complications following chlamydia infection are hampered by the unknown impact of unmeasured confounders including undiagnosed repeat chlamydia infections, other incident STIs and diagnostic biases”.
Interpretation	20	Give a cautious overall interpretation of results considering objectives, limitations, multiplicity of analyses, results from similar studies, and other relevant evidence	Consider alternative explanations of observed risk in:<br>Discussion, paragraph 2: “The absolute difference was 0.14%. There are several factors that have the potential to contribute to this observed excess risk of PID following a negative non-NAAT compared to a NAAT.“;<br>Discussion, paragraph 3: “This is broadly in keeping with the reported 65-75% sensitivity of non-NAATs compared to NAATs.”;<br>Discussion, paragraph 7“This estimate is remarkably consistent with the most robust observed risk of 9.46% (2.79-16.13) from the POPI RCT.”
Generalisability	21	Discuss the generalisability (external validity) of the study results	Discussion, paragraph 12: “Generalisation of the findings from this analysis to the contemporary setting may be compromised if intervening updates to chlamydia testing policies have led to a change in the composition (demographic or risk) of the population being tested for chlamydia.”
**Other information**			
Funding	22	Give the source of funding and the role of the funders for the	Included

	present study and, if applicable, for the original study on which the present article is based	

*Give information separately for exposed and unexposed groups.

Note: An Explanation and Elaboration article discusses each checklist item and gives methodological background and published examples of transparent reporting. The STROBE checklist is best used in conjunction with this article (freely available on the Web sites of PLoS Medicine at http://www.plosmedicine.org/, Annals of Internal Medicine at http://www.annals.org/, and Epidemiology at http://www.epidem.com/). Information on the STROBE Initiative is available at http://www.strobe-statement.org.
